# Modern Practice Era: The Purpose of Physician's Sample

**DOI:** 10.4103/0970-0218.45378

**Published:** 2009-01

**Authors:** Sugam A Bhatnagar, Jagannath V Dixit

**Affiliations:** Department of PSM, Government Medical College and Hospital, C 3 Asawari, Aurangabad, Maharashtra, India

## Introduction

Physician practice has evolved by a quantum in the last 20 years in India. With the mushrooming of state-of-the-art hospitals and multi-specialty clinics, the number of conventional self drug-dispensing ‘neighborhood’ clinics has nose dived lately. The act of giving medications in ‘Pudiyas’ (tablets wrapped in paper or plastic envelopes) was more a routine than just a practice by these neighborhood clinics. Physicians used to provide patients with unlabelled medications from their clinics as well as there used to be insistence on the part of the patient for medication from the doctor himself.

This practice of self drug-dispensing has become rare also owing to bubbling of multiple ‘Pharmacy Shops’ in close vicinity of these hospitals.

All these factors have brought an evolution in the methods and principles of medical practice and patient care and thus limited the use of physician sample which brings us to ask a very newfangled query ‘What is the purpose of physician's sample (PS)?’.

Just in the year of 2003, the pharmaceutical industry in the USA spent a staggering $16.4 billion(1) on distributing free samples through promotional face-to-face activities and as many as 78% American doctors accepted PS in the year 2007.(2) The physician's sample are free samples, though the cost of which are added to the drug formulation cost which is eventually borne by the patient. Thus, the patient becomes a victim of a practice which seems to serve limited function and provide little satisfactory reasoning. With advertising highly restricted in our country, this expenditure on a proportional basis may amount to a huge sum in our country as well.

The present study is conducted with the objective of finding out the purpose of PS as perceived by the three main stake holders: the physicians, medical representatives (MR) and the pharmaceutical company executives concerned with production of drugs and quality assurance.

## Materials and Methods

### Study design

In our attempt to understand the true purpose of PS in the context of modern practice era, we devised a 3 perspective study involving practicing doctors, medical representatives and executives of the pharmaceutical industry. The study was carried with the help of interview schedule in the 3 month period of June-July-August 2007. The interview schedule was pre-tested and then used.

### Sample selection and survey design

The sample of doctors was chosen from the resource of the Indian Medical Association (IMA) member list of Aurangabad and out of the 384 members, 38 doctors were selected on the basis of *systematic random sampling.* The doctors identified were from different specialties including internal medicine, paediatics, orthopaedics, surgery, dermatology, obstetrics and gynaecology, ophthalmology, ENT, radiology, psychiatry and general practice. They were subjected to a one-to-one interview. The interview took about 20 minutes.

10 MR were randomly chosen on the basis of their departments and divisions. They were selected when they were visiting and meeting doctors from different medical specialties. It was carefully screened that the MR met the doctors for providing free samples only and not for any other incentive.

4 executives from 4 of the 8 prominent pharmaceutical companies were chosen randomly for interview. These included production managers, quality assurance managers and supply managers.

## Result

Characteristic qualitative analysis of the respondents was done after the completion of survey and collection of data from the sample. Responses of the interviewees are shown below in the tables [Tables 1 and 2] present the responses of interviewees.

**Table 1:** Age, sex, and occupation wise distribution of interviewees

	Sex		Profession		
		
Age (years)	M	F	Dr.	MR	Exec
<30	9	2	4	7	0
31 to 45	20	7	23	3	1
46 onwards	12	2	11	0	3
Total	41	11	38	10	4

There are 38 doctors, 10 MR and 4 company executives included in the study. Most of the doctors are in the age group of 31 to 45 years and most of the MR are of less than 30 years of age

**Table 2:** Major purposes of physician's sample according to interviewees of all the 3 groups

Purpose*	No. of Doctors (38)	No. of MR (10)	No. of Exec. (4)
Poor patients	29	7	1
Promoting brand	18	5	4
For doctors feedback	5	2	3
Camps and social work	4	5	0

* One interviewee can give more than one response

## Discussion

Our data shows that the purpose of PS was more inadequate than the justification of its valid use. Suggesting that 76% doctors believed that main purpose of PS is to serve the poor patients, 86% of whom agreed that the purpose is not served effectively. While almost all the doctors used to get regular visits and PS, only 45% of the doctors accepted the PS. Most of the other 55% doctors believed that underutilization is the most important factor in their denial of acceptance. As many as three-quarters’ of the doctors believed that the practice is ineffective in serving the purposes it ought to. Several of the interviewees highlighted that free samples are dependent on the ‘pen power’ or ‘scalpel power’ of the doctor in question.(3) Interviewees strongly believed that when a seller whose basic objective beyond any shade of doubt is just to promote sales, becomes the educator, rationality is the first casualty.(3) This is amply evident in the study by Zweiffler *et al*, which states that patients with hypertension that are treated with free drug samples are less likely to have their hypertension controlled than are patients whose hypertension is treated by the physicians’ free choice of drugs.(3,4)

Pharmaceutical Company managers in their interview suggested that the main purpose of the PS is to create awareness about the brand name. Many of them suggested that reception of feedback from the doctor on the performance of drug boosts their R and D and is important part of the Clinical Trial stage IV. On the contrary, only 1 out of the 4 managers interviewed believed that the practice is ‘ethically skewed’. The exact manufacturing practices of PS could not be inquired but it was based on strategies of marketing and promotion, targeted mostly at doctors with higher ‘pen power’.

An objection put forth by most MR was relating to staff's or doctor's habit of taking sample drugs home rather than giving to the patients.(5) They also believed that a national authority for screening and monitoring of promotional data would be effective in a large way to model ethically the system of provision of free samples. Though all the MR believed that there is wastage of the PS, 7 out of 10 believed it is serving the purpose and some sort of underutilization is inevitable. They suggested that utilization of more than 50% of the sample is an indicator of efficient use. On the other hand, 2 out of 10 MR suggested that most doctors do not accept PS and the practice will be out of fashion sooner than later, they also believed that PS is a waste of raw materials, efforts, and environment. More than 50% believed that the ideal way to utilize these samples is to organize camps or donate to trusts; they also believed sample utilization is more effective in government institutions than private clinics.

## Conclusion

This conflicting multi-perspective approach has mainly arisen due to slow or probably no evolution in the practice of giving physician's sample (PS) while the principles of medical practice have changed substantially. We believe that this practice offers limited purpose in the modern medical practice scenario. On further critical analysis, we suggest that there should be a national debate among the stake holders including the patients to look into this matter as it is concerned directly with the pubic interests and money. Further rules and regulations should be established to prevent any unethical or unlawful way of business. An initiative taken by professional bodies of doctors, MR and consumers looking into information and *modus operandi* related to the distribution of PS would go a long way in preventing malpractices if any.
